# An MRI-based joint model of radiomics and spatial distribution differentiates autoimmune encephalitis from low-grade diffuse astrocytoma

**DOI:** 10.3389/fneur.2022.998279

**Published:** 2022-11-03

**Authors:** Sirong Piao, Xiao Luo, Yifang Bao, Bin Hu, Xueling Liu, Yuqi Zhu, Liqin Yang, Daoying Geng, Yuxin Li

**Affiliations:** ^1^Department of Radiology, Huashan Hospital, Fudan University, Shanghai, China; ^2^Institute of Functional and Molecular Medical Imaging, Fudan University, Shanghai, China; ^3^Academy for Engineering and Technology, Fudan University, Shanghai, China

**Keywords:** autoimmune encephalitis, low-grade diffuse astrocytoma, MRI, radiomics, spatial distribution, imaging analysis

## Abstract

**Background:**

The differential diagnosis between autoimmune encephalitis and low-grade diffuse astrocytoma remains challenging. We aim to develop a quantitative model integrating radiomics and spatial distribution features derived from MRI for discriminating these two conditions.

**Methods:**

In our study, we included 188 patients with confirmed autoimmune encephalitis (*n* = 81) and WHO grade II diffuse astrocytoma (*n* = 107). Patients with autoimmune encephalitis (AE, *n* = 59) and WHO grade II diffuse astrocytoma (AS, *n* = 79) were divided into training and test sets, using stratified sampling according to MRI scanners. We further included an independent validation set (22 patients with AE and 28 patients with AS). Hyperintensity fluid-attenuated inversion recovery (FLAIR) lesions were segmented for each subject. Ten radiomics and eight spatial distribution features were selected *via* the least absolute shrinkage and selection operator (LASSO), and joint models were constructed by logistic regression for disease classification. Model performance was measured in the test set using the area under the receiver operating characteristic (ROC) curve (AUC). The discrimination performance of the joint model was compared with neuroradiologists.

**Results:**

The joint model achieved better performance (AUC 0.957/0.908, accuracy 0.914/0.840 for test and independent validation sets, respectively) than the radiomics and spatial distribution models. The joint model achieved lower performance than a senior neuroradiologist (AUC 0.917/0.875) but higher performance than a junior neuroradiologist (AUC 0.692/0.745) in the test and independent validation sets.

**Conclusion:**

The joint model of radiomics and spatial distribution from a single FLAIR could effectively classify AE and AS, providing clinical decision support for the differential diagnosis between the two conditions.

## Introduction

Autoimmune encephalitis (AE) is a severe autoimmune mediated neuroinflammatory disorder that causes various neuropsychiatric syndromes, including seizures, behavior or cognitive dysfunction, or autonomic instability (1). Effective and timely immunotherapy is essential for a better prognosis. Currently, the diagnosis of AE relies on antibody testing and the treatment response to immunotherapy, which is limited by the availability of the test and treatment (1, 2). Therefore, early diagnosis of AE for more appropriate clinical management remains a significant challenge.

Although clinical manifestations provide crucial information for the diagnosis of AE, with the growing number of non-infectious cases identified in the last decade, radiological imaging, and MRI plays an increasingly critical role in the early diagnosis of AE (3). The typical MRI characteristics of AE include hyperintensities on T2-weighted images (T2WI) or fluid-attenuated inversion recovery (FLAIR) imaging in the medial aspect of the temporal lobes (1, 3).

However, AE shares common MRI characteristics with low-grade diffuse astrocytoma, a primary malignant brain tumor that usually requires surgical treatment (4). These shared features include hyperintensities on T2-weighted images (T2WI) without enhancement on post-contrast T1-weighted images (T1WI) (5). The misdiagnosis of the two conditions could result in unnecessary immunotherapy or surgical treatment (1, 6). Accurately differentiating the two conditions clearly impacts patient management and benefits patient quality of life.

The existing knowledge of differentiating AE from low-grade diffuse astrocytoma is largely based on sporadical case reports (5, 7–10). The previous studies demonstrated several characteristics in distinguishing autoimmune encephalitis from glioma through conventional MRI, and unilateral lesions and loss of differentiation between gray and white matter are also supporting signs for gliomas (5). However, subjective identification depends more on the clinical experience of the neuroradiologists. Thus, differential diagnosis remains a challenge in clinical practice. In addition, advanced MR sequences might be helpful to the situation (11–14), but their clinical application was limited due to complex processing steps. As a result, a more efficient and specific tool is needed to tackle the challenge of differentiating AE and glioma.

Radiomics is an emerging field that converts medical imaging to quantitative features using computational methods (15). Radiomics features, including morphological, statistical, and textural features, could provide information reflecting underlying pathophysiology that is difficult to capture by visual inspection (16). Recently, radiomics has been successful in the differential diagnosis of neurological diseases for clinical decision-making (17–20). Meanwhile, previous radiomics models (21, 22) also showed promising performance based on a single sequence. Particularly, FLAIR is a sequence widely used in routine clinical practice, which has demonstrated validated efficacy in characterizing both tumor core and peri-tumor edema. Therefore, radiomics based on the FLAIR sequence could provide essential information in differentiating the two diseases.

Scanty studies focused on differentiating neuroinflammation from low-grade glioma using computational methods (22). A previous study conducted a radiomics analysis to discriminate neuroinflammation from grade II glioma (22). This study established the feasibility of radiomics in differentiating AE from low-grade glioma. However, a model based on traditional radiomics may not be comprehensive enough to evaluate brain lesions based on relatively small sample size.

While radiomics features reflect the local information of the lesions, the global information could also be shown by the spatial distribution signatures, which provide unique information for characterizing glioma and AE. For instance, gliomas tend to be located in regions enriched with the genes associated with chromatin organization and synaptic signaling (23). Besides, it is reported that IDH-mutated low-grade gliomas are more located in the frontal lobes, while IDH wild type is more in the basal ganglia of the right hemisphere (24). As for AE, the lesion tends to spatially affect the limbic system (1, 25). Therefore, adding the spatial distribution features to the traditional radiomics features promises to better differentiate AE from low-grade glioma.

In this study, we hypothesized that a joint model of radiomics and spatial distribution features could improve the differentiation model between the two diseases. To test this hypothesis, we retrospectively collected 188 patients with a confirmed diagnosis of autoimmune encephalitis or low-grade astrocytoma. We then constructed a joint model based on the radiomics and spatial distribution features extracted from the FLAIR sequence. The model performance was validated in multiple imaging sets and also compared with the diagnostic performance of neuroradiologists.

## Materials and methods

**Figure 1:**
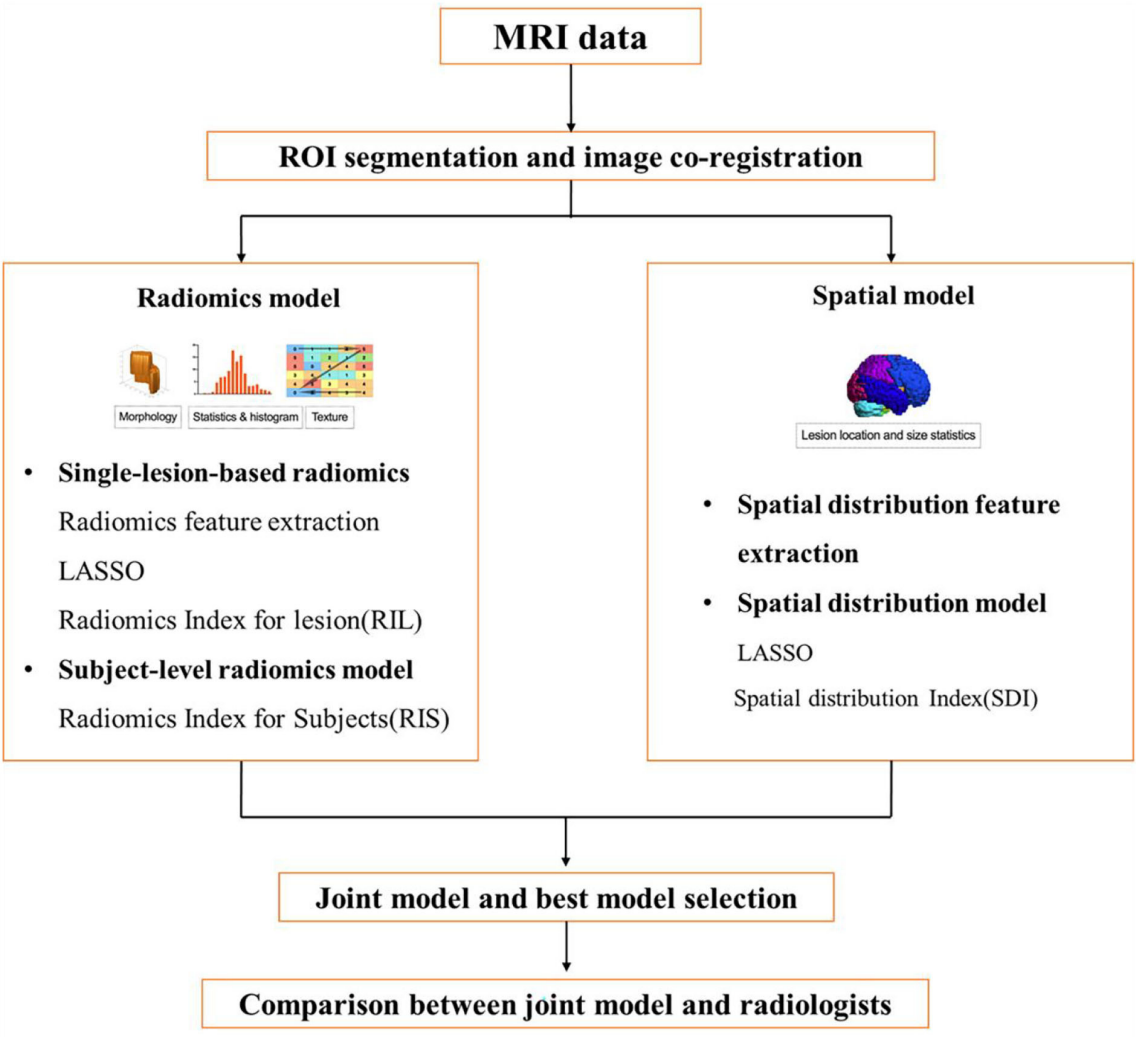
A flowchart of the study design.

The study was approved by the Institutional Review Board of Huashan Hospital, and the need for written informed consent was waived due to the retrospective nature of the study. All human studies were approved by the Huashan Hospital Ethics Committee and were performed following the ethical standards laid down in the 1964 Declaration of Helsinki and its amendments. The flowchart of the study design is shown in Figure 1.

### Patients

We included two cohorts with patients diagnosed with astrocytoma (AS cohort) or autoimmune encephalitis (AE cohort) from 2014 to 2021.

The inclusion criteria for the AE cohort were as follows: (1) a clinical diagnosis of AE according to the 2016 diagnostic criteria (1) by two neurologists; (2) positive laboratory examination for autoimmune antibodies (Abs), including neural surface antigens (NMDAR, LGI1, CASPR2, GABABR, and AMPAR) in both immunohistochemistry and cell-based assays for Abs, and dot-blot analysis for the presence of onco-neuronal Abs (anti-Hu, Yo, CV2, Ri, Ma2, and amphiphysin); and (3) availability of MRI in the acute onset, including T1WI, T2WI, FLAIR, diffusion-weighted images (DWI), and contrast-enhanced T1-weighted (CE-T1WI) images.

The inclusion criteria for the AS cohort were as follows: (1) histopathologically confirmed grade II astrocytoma (WHO 2016) (26); (2) availability of preoperative MRI, including T1WI, T2WI, FLAIR, DWI, and CE-T1WI; and (3) no history of brain tumors, brain biopsy, or other preoperative treatment.

We excluded the following cases for both cohorts: (1) previous history of neuropsychiatric diseases; (2) missing sequences; and (3) poor imaging quality. We also excluded the cases without apparent lesions on T2WI or FLAIR for the AE cohort, and the cases with representative manifestations for the AS cohort (space-occupying effect and heterogeneous signals, e.g., hemorrhage, necrosis, and cyst) with the consensus of two experienced neuroradiologists (Hu B. and Li YX.) blinded to the radiomic analysis.

### The training, test, and independent validation set

The patients diagnosed from 2014 to 2020 were randomly split into training and test sets at a 3:1 ratio, by stratified sampling according to the MRI scanner within each disease cohort using the R software (Version 3.6.2, http://www.r-project.org/), resulting in 103 patients in the training set (AE cohort: 44; AS cohort: 59) and 35 patients in the test set (AE cohort: 15, AS cohort: 20). The patients diagnosed in 2021 were taken as the independent validation set, including 22 patients with AE and 28 patients with AS.

### MRI protocols

MRI examination of the enrolled patients was performed on three 3.0T MRI scanners (two GE Discovery 750 scanners and one Siemens Viero MR scanner) using the head coil with eight channels.

The following sequences were included as follows: T1WI, T2WI, FLAIR, DWI, and CE-T1WI acquired after intravenous injection of 0.1 mmol/kg of gadolinium-chelate contrast medium. The scan parameters on GE MR750 were shown as follows: T1WI (repetition time, TR 2,000 ms, echo time, and TE 9 ms), T2WI (TR 3,000 ms and TE 98 ms), and FLAIR (TR 7,000 ms, TE 93 ms, and TI 2,100 ms). The scan parameters on Siemens Viero MR included the following: T1WI (TR 2,000 ms and TE 17 ms), T2WI (TR 4,552 ms and TE 97 ms), and FLAIR (TR 8,525 ms, TE 142 ms, and TI 2,501 ms). The slice thickness of all the axial images was 5 mm with a 1 mm interslice gap, and the field of view was 240 × 240 mm. Digital imaging and communications in medicine (DICOM) data were converted to NIfTI format for later processing, and then the basic information of patients was simultaneously removed for the protection of privacy.

### Region of interest (ROI) segmentation

*The region of interests* of each scan of each subject were segmented manually on FLAIR images. For AS cases, the ROI covered the enhancing tumor and peripheral edema and was adjusted following the inspection of DWI and CE-T1WI images. For AE cases, the ROI covered all the hyperintense T2 lesions referring to T1WI and DWI images; single lesions with a maximal in-plane length of fewer than five voxels (<2.5 mm) were ignored.

The ROIs were manually drawn by a neuroradiologist with 4 years of experience (Piao SR.). The consensus was reached after a careful review and modified by an experienced neuroradiologist (Li YX, with 20 years of experience in neuroradiology). Both neuroradiologists were blinded to the diagnosis and grouping of the patients. A segmentation threshold and region-growing segmentation algorithm implemented using the software (MITK; www.mitk.org) were used to create these ROIs.

### Construction and evaluation of the radiomics model

#### Radiomics features extraction and quantification

Radiomic features were extracted from each lesion ROI using the Standardized Environment for Radiomics Analysis (SERA) (27–29) (https://qurit.ca/software/sera/) implemented in MATLAB (Version 2020b; MathWorks). According to guidelines from the Image Biomarker Standardization Initiative (IBSI) (30), 351 radiomics features (29 morphology, 20 statistics, 30 histogram features, and 272 higher-order texture features) were included for the following analysis.

#### Radiomics feature selection

All extracted radiomics features were standardized before feature selection. Spearman's correlation matrix of the features was first calculated. Subsequently, hierarchical clustering was performed based on the correlation matrix, selecting the most representative features.

Next, the selected features were input to a least absolute shrinkage and selection operator (LASSO) (29), to build a radiomics signature for discriminating AS and AE. The classifier was trained using 10-fold cross-validation on the training set to determine the optimal parameters among the full coefficient paths, where the most predictive features and their weights were determined. A Radiomics Index for Lesion (RIL) was calculated by summing the selected features and corresponding weights from the single-lesion-based radiomics model. A threshold *T* was defined as the optimal cutoff by the maximum Youden index in receiver operating characteristic (ROC) curves, classifying each lesion into negativity-like (RIL<T) or positivity-like lesion court (RIL≥T).

#### Subject-level discrimination model

To optimally merge RIL-based single-lesion classification into a subject-level diagnosis, a lesion-merged function (30) was used for defining and calculating the Radiomics Index for Subject (RIS). By comparing the summated distances of RIL^−^ court (negativity-like) and RIL^+^ court (positivity-like) lesions relative to the threshold *T*, the court with a higher summated distance was selected as the diagnosis of a scan, and the distance of the corresponding court was averaged as the RIS. With *n* and *p* denoting the numbers of all lesions in RIL^−^ court (negativity-like lesions with RIL<*T*) and RIL^+^ court (positivity-like lesions with RIL>*T*), respectively, and the RIS value of each scan is calculated as the following expression:


$$RIS=\{\begin{array}{c} \frac{1}{p}\sum_{i=1}^{p}RIL_{i}^{+},\sum_{i=1}^{p}|RIL_{i}^{+}-T|\geq \sum_{j=1}^{n}\left|RIL_{j}^{-}-T\right|\;\\ \frac{1}{n}\sum_{j=1}^{n}RIL_{j}^{-},\sum_{i=1}^{p}|RIL_{i}^{+}-T|<\sum_{j=1}^{n}\left|RIL_{j}^{-}-T\right| \end{array}$$

### Construction and evaluation of the spatial distribution model

Spatial distribution feature extraction was processed through probabilistic lesion mapping procedures *via* SPM12 (http://www.fil.ion.ucl.ac.uk/spm) (31). First, the T1W image was co-registered with the T2-FLAIR image. Next, the converted T1W image was transformed into the Montreal Neurology Institute (MNI) space. Then, the transformation parameter was applied to the FLAIR image and the whole-brain lesion ROI. Finally, the threshold of each lesion mask in the standard space was set to 0.5 and binarized to avoid the expansion of the lesion caused by normalization.

After those above pre-processing, 27 spatial distribution features were extracted based on the MNI-space lesion masks for each scan, including lesion location distribution statistics represented by the number of lesion voxels in different brain regions (12 supratentorial and four infratentorial) and lesion size distribution statistics including the number of lesions, mean/maximum/summed sizes, and the number of lesions within seven size ranges (8~80, 80~160, 160~320, 320~640, 640~1,280, 1,280~2,560, and >2,560 mm^3^).

The spatial distribution model was constructed using a similar LASSO method as the radiomics model, with the spatial distribution features of each scan as inputs. The output of the spatial distribution model was named as spatial distribution index (SDI) for each scan.

### Joint model and model selection

A joint radiomics and spatial distribution model (hereinafter referred to as joint model) was constructed as the combination of RIS from the radiomics model and SDI from the spatial distribution model by logistic regression.

The performance of the radiomics model, the spatial distribution model, and the joint model was compared by the ROC curve. The corresponding AUC, accuracy, sensitivity, and specificity of the models were evaluated in the training and test sets and additionally assessed in the independent validation set.

### Comparing diagnostic performance with neuroradiologists

Two neuroradiologists (Bao YF and Zhu YQ, with 12 and 4 years of experience, respectively) independently reviewed all 188 cases. Both were blinded to the final diagnosis and grouping and judged purely based on the MRI and clinical information. The diagnostic agreement of the two neuroradiologists was assessed and compared with the joint model.

### Statistical analysis

All statistical analyses were performed using the R software and GraphPad Prism 8.0. The clinical characteristics of the two cohorts were compared using Fisher's exact test for nominal categorical variables and an independent *t*-test for continuous variables. The Kappa test was used to assess the inter-observer agreement of the two neuroradiologists. The ROC analysis was performed to compare the performances of radiomics, spatial signatures, and the neuroradiologists' assessment. The AUCs of the different models were compared using the DeLong test. A two-sided *P* < 0.05 was considered significant.

## Results

### Patient characteristics

Detailed patient characteristics are shown in Table 1. No significant difference was found in demographic characteristics between the AE and AS groups. In the AE group, the types of the auto-antibodies were as follows: AMPA2 (*n* = 1), CASPR2 (*n* = 1), CV2 (*n* = 1), DPPX (*n* = 1), GABA (*n* = 9), GAD (*n* = 7), LGI1 (*n* = 18), MA2 (*n* = 1), MOG (*n* = 2), NMDA (*n* = 33), PNMA2 (*n* = 1), and multiple/co-existing auto-antibodies (*n* = 6). They mainly showed extensive lesion distribution, and most were in the temporal lobe. Some showed leptomeningeal enhancement on post-contrast T1WI.

**Table 1:** The characteristics of different data sets.

**Characteristics**	**Overall (*n* = 188)**	**Training set**	**Test set**	**Independent validation set**	***p*-value**
Age (years)	41.36 ± 15.54	41.19 ± 15.13	39.49 ± 15.98	44.10 ± 15.31	0.599
Gender					
Male	125	68	23	34	1
Female	63	35	12	16	
Disease type^†^					
AE	81	44	15	22	-
AS	107	59	20	28	
Total number	188	103	35	50	

Ages are mean ± standard deviation, and others are the number of patients. The p-values are generated from the comparisons of the characteristics between training and test sets.

† AE: definite autoimmune encephalitis, AS: WHO grade II astrocytoma confirmed by histopathology.

In the AS group, seven subjects (6.54%) exhibited multiple lesions located on bilateral hemispheres. The rest were single lesions in either the left hemisphere (54/107, 50.47%) or the right hemisphere (46/107, 42.99%). For lesion location, 56 subjects were found with lesions in the frontal lobe, 31 in the temporal lobe, eight in the parietal lobe, five in the insula, and other locations, mainly including the occipital lobe, thalamus, and brain stem. Thirty-three subjects showed enhancement on CE-T1WI, 11 of which were significant enhancement, and others were mild-to-moderate enhancement. Besides, 21 subjects were found to have T2-FLAIR mismatch manifestation or showed rim on FLAIR.

### Performance of the radiomics model

**Figure 2:**
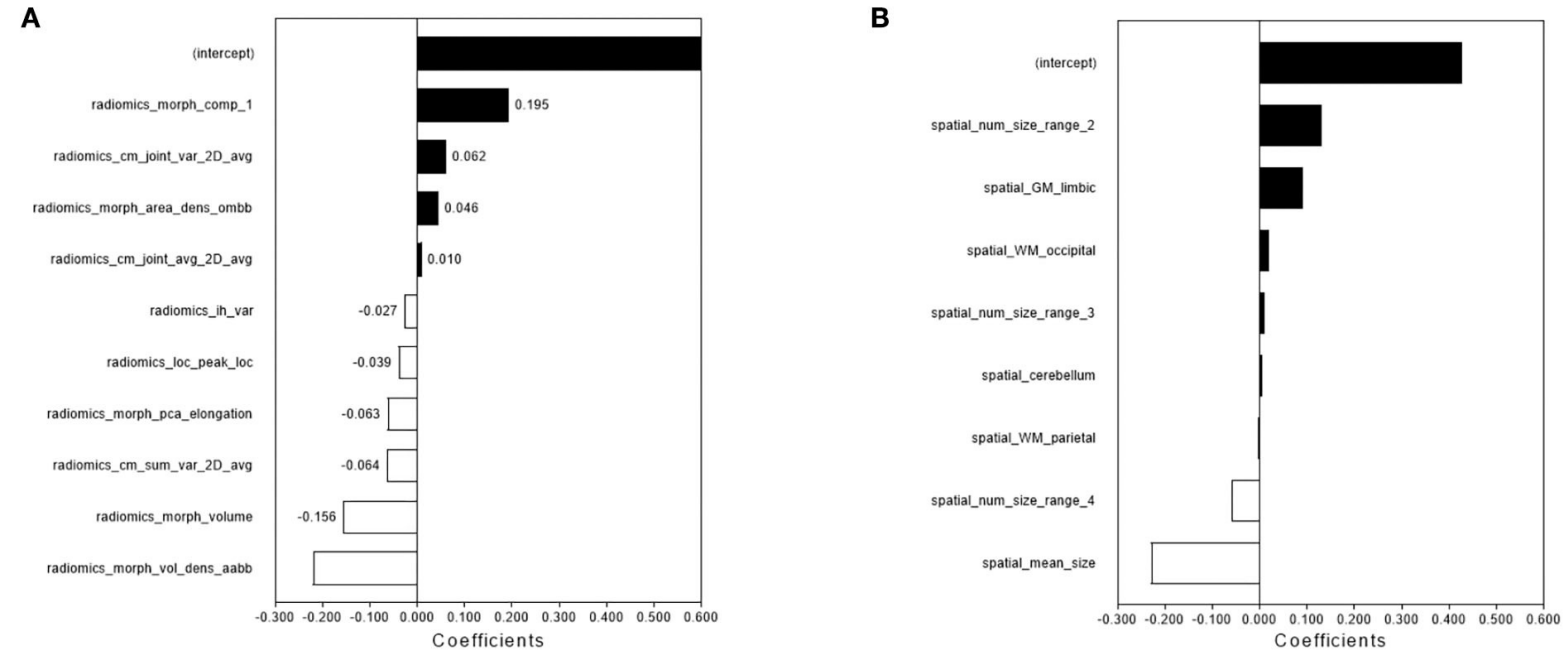
The feature selection and performance of the radiomics model and spatial distribution model. The radiomics features **(A)** and spatial distribution features **(B)** were selected by LASSO and the selected features were trained for the establishment of the single-lesion-based radiomics model and spatial distribution model.

Ten radiomics features were selected to construct a radiomics classifier (Figure 2), which achieved comparable performances on the training (AUC 0.964, accuracy 0.951, sensitivity 0.977, and specificity 0.932) and the test set (AUC 0.910, accuracy 0.947, sensitivity 0.933, and specificity 0.957). There were no significant differences in the performance of the training and the test set (*p* > 0.05).

### Performance of the spatial distribution model

Eight features were selected to construct a spatial distribution classifier (Figure 2), which also achieved comparable performances on the training (AUC 0.983, accuracy 0.951, sensitivity 0.932, and specificity 0.966) and the test set (AUC 0.930, accuracy 0.943, sensitivity 1.000, and specificity 0.900). No significant difference was found in the performance of the training and test sets (*p* > 0.05).

### Performance of the joint model

The logistic regression model, which incorporated RIS (coefficient = 12.899, *p* = 0.005) and SDI (coefficient of SDI=17.735, p=0.003), achieved better performance than both separate models (training set: AUC 0.994, accuracy 0.951; test set: AUC 0.957, sccuracy 0.914). The model achieved slightly lower performance in the independent validation set, and the AUC, accuracy, sensitivity, and specificity were 0.908, 0.840, 0.964, and 0.929, respectively.

The DeLong test comparing the performance of the training, test, and independent validation sets showed no significant difference (training vs. test, *p* = 0.298; training vs. independent validation, *p* = 0.116; and test vs. independent validation, *p* = 0.453). In testing the effect of different image acquisition settings, Fisher's exact test showed no significant difference (*p* = 0.552, 0.733, and 1.000 for the training, test, and independent validation sets, respectively). Detailed information is available in the Supplementary Table.

### Comparing the models with neuroradiologists

The senior neuroradiologist (Bao YF) achieved consistently better performance (AUC: 0.932, 0.917, and 0.875) than the junior neuroradiologist (Zhu YQ, AUC: 0.661, 0.692, and 0.745) in diagnosing the training, test, and independent validation sets, respectively. The inter-observer agreement of the two neuroradiologists showed a Kappa value of 0.336, 0.394, and 0.618 in the training, test, and independent validation sets, respectively.

In addition, we compared the diagnostic performance of the two neuroradiologists with the joint model. In all three sets, the diagnostic performance of the joint model was significantly higher than the junior neuroradiologist (*p* < 0.0001 in the training set, p=0.001 in the test set, and p=0.013 in the independent validation set). In contrast, there is no statistical difference between the diagnostic performance of the joint model and the senior neuroradiologist in the test and independent validation set (both *p* > 0.05).

**Figure 3:**
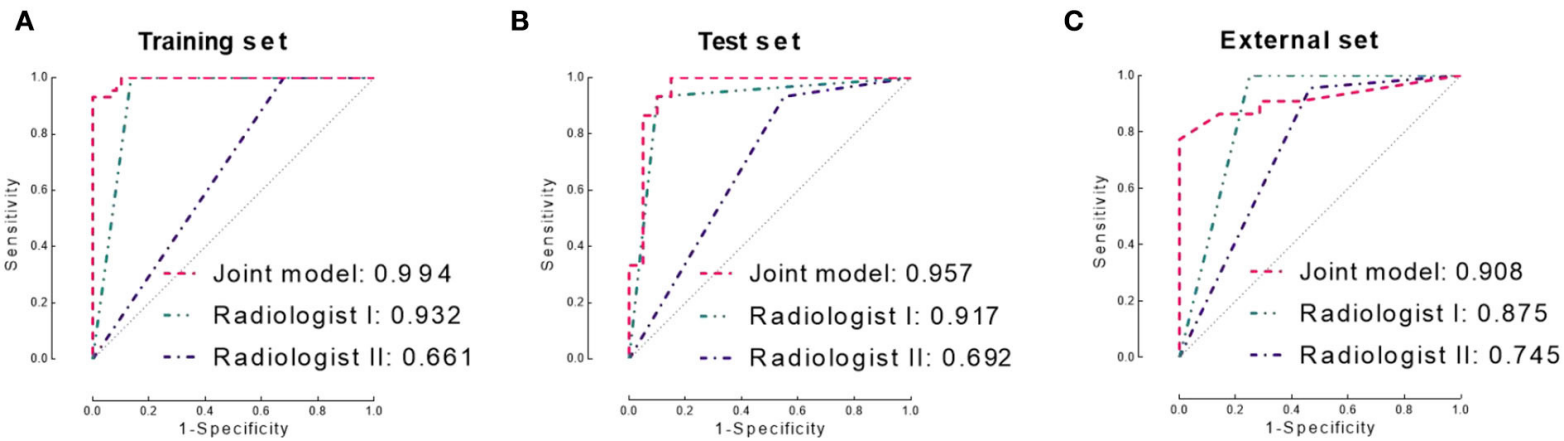
Receiver operating characteristic (ROC) curves of the joint model and two neuroradiologists in the training set **(A)**, test set **(B)**, and independent validation set **(C)**, respectively. AUC, area under the ROC curve.

**Figure 4:**
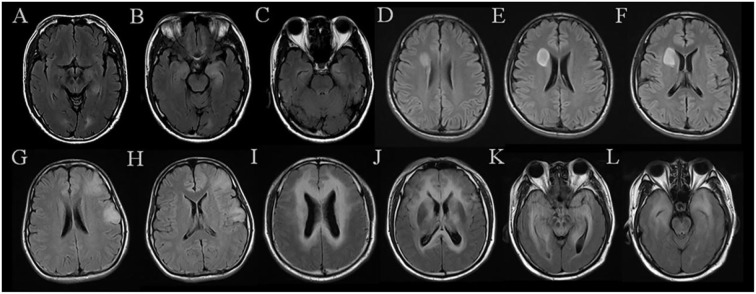
MR images of representative cases. **(A–C)** A case in the AE group shows symmetrical hyperintensities of the hippocampus on FLAIR. **(D–F)** A case in the AS group exhibits a mass located in the right frontal lobe with a quite clear boundary. **(G,H)** A case in the AS group was misdiagnosed as AE by two neuroradiologists, which shows patchy abnormalities located in the left frontal lobe and temporal lobe. **(I–L)** A case in the AS group shows multiple and symmetrical hyperintensities throughout the brain, which was diagnosed incorrectly by both neuroradiologists. AE, short for definite autoimmune encephalitis; AS, short for WHO grade II astrocytoma confirmed by histopathology.

The diagnostic performance of the three models is displayed in Table 2. The ROC curves are shown in Figure 3. MR images of representative cases are shown in Figure 4.

**Table 2:** The diagnostic performance of the three models.

	**Radiomics model**		**Spatial model**		**Joint model**	
	**Train**	**Test**	**Train**	**Test**	**Train**	**Test**
AUC	0.964	0.910	0.983	0.930	0.994	0.957
Accuracy	0.951	0.947	0.951	0.943	0.951	0.914
Sensitivity	0.977	0.933	0.932	1	0.932	0.867
Specificity	0.932	0.957	0.966	0.900	0.966	0.950

## Discussion

Our study proposed a novel radiomics-based approach for distinguishing AS from AE. The joint model based on radiomics and spatial distribution features achieved promising accuracy and similar performance with an experienced neuroradiologist, outperforming a junior neuroradiologist.

Currently, a large number of radiomics studies have been reported, especially in the field of brain tumors, covering a wider range of tasks, including differentiating subtypes (32–34), tumor grading (35), and predicting the prognosis (36, 37). The previous studies showed that first- or second-order texture features were particularly useful in distinguishing benign and malignant brain tumors (38, 39).

However, few studies tackled the challenge of discriminating brain tumors from neuroinflammation. Han et al. conducted a radiomics analysis classifying 39 patients with grade II glioma from 18 patients with neuroinflammation, which achieved the AUC of 0.950 and 0.925 based on multiple sequences. Interestingly, the performance based on a single sequence remained promising (22). In comparison, our joint model performed better than this study, which suggests the significance of integrating local and global information from radiomics and spatial distribution signatures. In our study, the spatial distribution analysis quantitatively characterizes the lesion distribution location and size of the lesion. The lesion location could account for the pathological pathways. The lesion size characteristics could also reflect the lesion distribution and disease severity. For example, different kinds of AE can also be different in the lesion distribution owing to the different nature of the auto-antibodies, such as MOG-IgG, which affected the integrity and function of the myelin through MOG protein, mainly localized on the outer surface of the myelin sheath and oligodendrocytes in central nervous system (31, 40). Furthermore, due to the minor interference caused by MR parameters, the spatial distribution model is more stable than the radiomics model, especially under the circumstance of a small sample size.

The FLAIR sequence in the present study achieved good performance, which suggested that the proposed computer-based approach has the potential to be applied based on the MRIs acquired in routine clinical practice. On FLAIR, the edema and ischemia reflected were exhibited obviously, and texture features extracted from FLAIR potentially provided more information reflecting the pathophysiological changes in lesions considered more obvious on FLAIR, which could also be clues in the differentiation of neoplastic lesions and inflammatory diseases.

Our study has clinical significance as it provides an automatic approach to differentiate AE from glioma. Most importantly, our model achieved comparable performance with experienced neuroradiologists, which shows the potential to aid clinical decision-making in a cost-effective and time-efficient manner. The proposed approach based on quantitative features could provide a comprehensive and objective analysis of the MRI data, compared with the visual evaluation of neuroradiologists which sometimes could lead to bias.

There were several limitations in this study. Due to the retrospective nature of the study, different subtypes of AE mediated by different auto-antibodies were not separated due to the rarity of the condition. With more cases available, future studies could focus on the characteristics of AE with specific antibodies. Besides, our joint model of radiomics and spatial distribution was mainly based on the FLAIR sequence. The discrimination values of other sequences between brain tumors and inflammation will be analyzed in further study. Additionally, MRIs were acquired from multiple scanners. Although no significant influence was found from the acquisition settings, the results need to be further validated *via* a prospective multi-center study.

In conclusion, our joint model of radiomics and spatial distribution could provide an effective method for classifying autoimmune encephalitis and low-grade diffuse astrocytoma. The joint model from a single sequence on MRI exhibited a much more convenient and efficient process and protocols based on global and local signatures, with a clear path that is easy to follow in further practice.

## Data availability statement

The original contributions presented in the study are included in the article/Supplementary material, further inquiries can be directed to the corresponding authors.

## Ethics statement

The studies involving human participants were reviewed and approved by the Institutional Review Board of Huashan Hospital. Written informed consent from the patients/participants or patients/participants' legal guardian/next of kin was not required to participate in this study in accordance with the national legislation and the institutional requirements.

## Author contributions

YL: supervision, writing—review and editing, project administration, and conceptualization. DG: supervision and writing—review and editing. LY: methodology and software. YZ and YB: validation and visualization. XuL: investigation and visualization. BH: visualization and conceptualization. XiL: formal analysis, methodology, and software. SP: writing—original draft, data curation, and visualization. All authors contributed to the article and approved the submitted version.

## Funding

This work was funded by the National Natural Science Foundation of China (61672236), the Science and Technology Commission of Shanghai Municipality (20S31904300 and 20511101100), Shanghai Municipal Key Clinical Specialty (SHSLCZDZK03201), Shanghai Hospital Development Center (SHDC2020CR3020A), and Greater Bay Area Institute of Precision Medicine (Guangzhou) (KCH2310094).

## Conflict of interest

The authors declare that the research was conducted in the absence of any commercial or financial relationships that could be construed as a potential conflict of interest.

## Publisher's note

All claims expressed in this article are solely those of the authors and do not necessarily represent those of their affiliated organizations, or those of the publisher, the editors and the reviewers. Any product that may be evaluated in this article, or claim that may be made by its manufacturer, is not guaranteed or endorsed by the publisher.
