# Supplementary material for: An MRI-based joint model of radiomics and spatial distribution differentiates autoimmune encephalitis from low-grade diffuse astrocytoma
Source: Front Neurol. 2022 Nov 3;13:998279. doi: 10.3389/fneur.2022.998279 (PMC9669344; doi:10.3389/fneur.2022.998279)
Supplement: Supplementary file 1 [file Data_Sheet_1.docx]

**Supplemental Table 1 Numbers of cases from two MR scanners by stratified sampling**

|  | GE MR750 | Siemens Viero MR |
| --- | --- | --- |
| Training set |  |  |
| AS cases | 29 | 30 |
| AE cases | 22 | 22 |
| Test set |  |  |
| AS cases | 10 | 10 |
| AE cases | 8 | 7 |
| External set |  |  |
| AS cases | 10 | 10 |
| AE cases | 8 | 7 |

**Supplemental Table 2 Comparison of diagnostic performance of the joint model from two MR scanners by stratified sampling**

|  | GE MR750 | Siemens Viero MR |
| --- | --- | --- |
| Training set |  |  |
| AS diagnosed by joint model | 28 | 32 |
| AE diagnosed by joint model | 23 | 20 |
| Test set |  |  |
| AS diagnosed by joint model | 10 | 11 |
| AE diagnosed by joint model | 8 | 6 |
| External set |  |  |
| AS diagnosed by joint model | 15 | 14 |
| AE diagnosed by joint model | 10 | 11 |
